# Metabolic dynamics of human external urethral sphincter myoblast differentiation and the effects of tricarboxylic acid cycle inhibition

**DOI:** 10.1038/s41598-025-13764-z

**Published:** 2025-08-07

**Authors:** Hironori Kai, Shinro Hata, Noriko Hamamatsu, Mayuka Shinohara, Keiko Matsuura, Hiromitsu Mimata, Toshitaka Shin

**Affiliations:** 1 Department of Urology, Faculty of Medicine, Oita University, Yufu, Oita 879-5593 Japan; 2 Department of Biomedicine, Faculty of Medicine, Oita University, Yufu, Oita 879-5593 Japan

**Keywords:** Stress urinary incontinence, External urethral sphincter, Myoblast differentiation, Tricarboxylic acid cycle, Metabolomic pathways, Cell biology, Urology, Urethra

## Abstract

Stress urinary incontinence commonly arises with aging or following prostatectomy, yet its underlying mechanisms remain unclear. To address this, we investigated the role of metabolic pathways—particularly the tricarboxylic acid (TCA) cycle—in the differentiation of human external urethral sphincter myoblasts. Immortalized sphincter cells (US2-KD) were induced to differentiate over 192 h. Metabolomic profiling using gas chromatography–mass spectrometry, along with pathway enrichment analysis, identified key metabolic changes. Inhibition of mitochondrial pyruvate transport with UK5099 markedly suppressed TCA cycle metabolites, including citrate, α-ketoglutarate, fumarate, and malate. This inhibition also significantly reduced *MYH7* expression and intracellular adenosine triphosphate levels throughout the differentiation period. These results demonstrate that the TCA cycle plays a critical role in both energy metabolism and the differentiation of urethral sphincter myoblasts. This study is the first to suggest that impaired TCA cycle activity may contribute to the pathogenesis of Stress urinary incontinence and represents a potential therapeutic target. Our findings offer new insight into age-related metabolic decline associated with Stress urinary incontinence and support the development of therapies that combine metabolic modulation with regenerative approaches.

**Supplementary Information:**

The online version contains supplementary material available at 10.1038/s41598-025-13764-z.

## Introduction

Stress urinary incontinence (SUI) significantly affects the quality of life and commonly occurs with aging or after radical prostatectomy^1,2^. SUI is caused by damage to the pelvic floor muscles, nerves, and connective tissues^3^, leading to dysfunction of human external urethral sphincter (hEUS), a major contributor to this condition.

In recent years, local therapies such as platelet-rich plasma (PRP)^4^, myogenic progenitor cells^5^, stem cells^6^, and adipose-derived stem cells^7^ have been used to promote functional hEUS regeneration. Additionally, treatments using stem cell secretomes^8^ and mesenchymal stem cells^9^ have gained attention. These therapies aim to achieve functional recovery through diverse mechanisms, including the paracrine effects of cytokines and growth factors, stem cell multipotency, extracellular matrix remodeling, and tissue regeneration. Although stem cell–based therapies hold substantial promise, treatment outcomes vary depending on several factors, such as cell type, delivery method, and follow-up duration. Significantly, challenges remain in achieving long-term efficacy and standardizing protocols, highlighting the need for further research.

We previously described the primary culture of hEUS satellite cells and the effects of secretomes containing IGF-1^10^, HGF^11^, and TNF-α^12,13^ on hEUS proliferation and differentiation. IGF-1 promotes myogenic differentiation of hEUS satellite cells via activation of the PI3-K pathway, whereas TNF-α inhibits their differentiation. HGF does not induce myogenic differentiation and may even suppress it during the differentiation phase. Nevertheless, the comprehensive differentiation mechanisms and metabolic pathways underlying functional regeneration remain poorly understood, posing significant barriers to the development of novel therapeutic strategies.

External urethral sphincter is developmentally and anatomically distinct from typical skeletal muscles, with some studies suggesting its partial origin via transdifferentiation from smooth muscle—an unusual feature among skeletal muscles^14^.

To address these challenges and establish new treatment approaches for SUI, we conducted a comprehensive analysis of metabolites in the culture supernatant of hEUS myoblasts and investigated the metabolic pathways involved in hEUS myoblast differentiation. Preliminary metabolomic analysis revealed dynamic alterations in the levels of tricarboxylic acid (TCA) cycle intermediates, indicating their critical roles in regulating differentiation processes. Furthermore, age-related decreases in TCA cycle function^15^ are associated with increased incidence of SUI in older populations. Therefore, we focused on the central role of the TCA cycle in energy metabolism and investigated its relationship with hEUS myoblast differentiation. By targeting the TCA cycle function using existing drugs or agents, we aim to develop novel therapeutic strategies for SUI.

## Materials and methods

### Ethics information

All procedures performed in studies involving human participants were in accordance with the ethical standards of the institutional and/or national research committee and with the 1964 Helsinki Declaration and its later amendments or comparable ethical standards. This study was approved by the institutional review board of Oita University (approval number: 307). Written informed consent was obtained from all participants.

### Cell culture

The establishment of the immortalized male human external urethral sphincter cell line (US2-KD) used in this study has been previously reported^13^. Briefly, primary myogenic cells were isolated from human external urethral sphincter tissue and immortalized by introducing mutated cyclin-dependent kinase 4, cyclin D1, and human telomerase reverse transcriptase genes, which enabled long-term culture and maintenance of myogenic differentiation potential. US2-KD cells were initially seeded in six-well plates coated with type I collagen (Corning Life Sciences, Corning, NY, USA) at a density of 1 × 10^5^ cells per well. The cells were cultured in growth medium (GM) consisting of high-glucose (4.5 g/L) Dulbecco’s modified Eagle’s medium (DMEM, Thermo Fisher Scientific, Waltham, MA, USA) supplemented with 20% fetal bovine serum (FBS; Sigma-Aldrich, St. Louis, MO, USA) and 2% Ultroser G serum substitute (Cat. no. 15950-017, Cytiva, Marlborough, MA, USA) for the first 48 h (h). This time point, termed the Pre-state, marks the end of the initial growth phase before differentiation induction. At this point, GM was replaced with differentiation medium (DM) containing high-glucose DMEM, 2% FBS, 5 µg/mL holotransferrin, 0.5 µg/mL insulin, and 10 nmol/L selenite. Cultures were maintained at 37 °C in a humidified atmosphere of 5% CO_2_. Subsequently, DM was replaced at 96, 144, and 192 h after seeding.

Sampling was performed every 48 h (at 48, 96, 144, and 192 h after induction of differentiation). This interval was chosen based on preliminary experiments showing that both morphological changes and the expression of the myogenic marker MYOG became prominent at 48-hour intervals during myoblast differentiation. Sampling every 24 h was avoided because it imposed excessive mechanical and metabolic stress on the cells, which led to reduced viability and reproducibility. Conversely, longer intervals risked missing important temporal changes in differentiation. Therefore, 48-hour intervals allowed us to accurately capture the differentiation process while maintaining cell health and experimental consistency.

### Quantitative real-time (qRT)-PCR

After collecting the culture supernatant for metabolomic analysis, cells were harvested in biological triplicate for RNA extraction. qRT-PCR was performed using the LightCycler system (Roche Diagnostics, Indianapolis, IN, USA) to evaluate the expression of MYOG (Cat no. QT00001722), MYH7 (Cat. no. QT00000602), and GAPDH (Cat. no. QT01192646). Gene expression was calculated using the ΔΔCt method, with GAPDH serving as the internal control. All primers for gene amplification were purchased from QIAGEN (Hilden, Germany).

### TCA cycle suppression medium preparation

To investigate the role of TCA cycle function in myoblast differentiation, mitochondrial pyruvate carrier (MPC) activity was inhibited using UK5099 (CAS No. 56396-35-1, Sigma-Aldrich)^16^. The 50 µM concentration was selected based on preliminary dose-response experiments (10–50 µM), which showed that lower concentrations (10–25 µM) sometimes promoted differentiation and resulted in inconsistent effects. Therefore, 50 µM was chosen to ensure reproducibility and stable inhibition of differentiation. No significant cytotoxicity was observed at this concentration (Supplementary Fig. S1).

US2-KD cells were cultured in GM until reaching subconfluence, at which point the medium was replaced with DM containing 50 µM UK5099 (final DMSO: 0.1%). Control cells received 0.1% DMSO without UK5099. UK5099 and DMSO were replenished every 48 h.

### Gas chromatography–mass spectrometry (GC–MS)

Metabolites in culture supernatant were analyzed using a GCMS-TQ8030 system (Shimadzu Corporation, Kyoto, Japan). Samples were prepared using a methanol–water–chloroform extraction method with 2-isopropylmalic acid as an internal standard. Chromatographic separation was performed using a DB-5 capillary column under standard GC–MS conditions, and metabolites were identified using the SHIMADZU Smart Metabolites Database. Peak integration and quantification were performed using GCMS solution ver.4.53 (Shimadzu Corporation), and the relative peak areas were calculated for each metabolite as an index of abundance. At each time point, culture supernatants were collected in triplicate (*n* = 3) from independently cultured wells. For the 0 h (Pre) time point, supernatants were collected immediately after replacing GM with DM; thus, Pre-supernatants represent DM-only controls. At each sampling time point, the culture supernatant was collected and centrifuged at 1,600 rpm for 5 min at 4 °C to remove detached cells and debris. The resulting supernatant was transferred to a new tube and stored at − 80 °C until analysis. All raw metabolite measurements and pathway enrichment results are available in Supplementary Table S1-5.

### Western blotting

Myotubes were lysed in Mammalian Protein Extraction Reagent (M-PER, Cat. no. 78501, Thermo Fisher Scientific) supplemented with protease inhibitors (Cat. no. 07575-51, FUJIFILM Wako Pure Chemical Corporation, Osaka, Japan). Protein concentrations were determined using the Bradford assay (Cat. no. 23200, Thermo Fisher Scientific). Equal amounts of protein (20 µg per lane) were separated on 4–20% Tris-glycine gels (Cat. no. 01-028-10, Tefco, Tokyo, Japan) and transferred to nitrocellulose membranes (0.45 μm, Cat. no. 1620115, Bio-Rad, Hercules, CA, USA). Membranes were blocked with 5% non-fat milk in TBST for 1 h at room temperature, followed by overnight incubation at 4 °C with anti-MYHC antibody (1:500, Cat. no. 05-716-I-25UL, MilliporeSigma, Burlington, MA, USA) and anti-β-actin antibody (1:10,000, Cat. no. HRP-60008, Proteintech, Rosemont, IL, USA). After washing, membranes were incubated with HRP-conjugated anti-mouse secondary antibody (1:10,000, Cat. no. 7074 S, Cell Signaling Technology, Danvers, MA, USA) for 1 h at room temperature. Signals were detected using ECL Prime (Cat. no. RPN2232, Cytiva). Due to limited protein yield, cells were cultured in triplicate and pooled for protein extraction at each time point. This procedure was independently performed twice per time point (*n* = 2 biological replicates). Each blot was quantified in technical triplicate using ImageJ (version 1.54 g, NIH, USA), and representative blots are shown in the figures. Western blot images were processed for brightness and contrast using Adobe Photoshop (ver.9.0, Adobe Inc., San Jose, CA, USA). All images were adjusted equally and no specific features were selectively enhanced or removed. Full, uncropped blots are provided in Supplementary Figure S2-1 and S2-2.

### Immunostaining

Cells were fixed with 100% methanol for 20 min at room temperature and permeabilized. After PBS washes, slides were blocked with 10% normal goat serum (Cat. no. 06-349-64, Nacalai Tesque, Inc., Kyoto, Japan) in PBS for 30 min. Slides were incubated with anti-MYHC antibody (Cat. no. 05-716-I-25UL, MilliporeSigma) overnight at 4 °C. Slides were then incubated with EnVision anti-mouse secondary antibody (Cat. no. K4001, Agilent Technologies, Dako Denmark A/S, Glostrup, Denmark) for 30 min at room temperature. Signals were developed with DAB substrate (Cat. no. K3468, Agilent Technologies, Dako Denmark A/Sk) for 10 min. Nuclei were counterstained with hematoxylin (Cat. no. 30002, Muto Pure Chemicals Co. Ltd., Tokyo, Japan) and mounted with CC/Mount (Cat. no. K002, Diagnostic BioSystems, Pleasanton, CA, USA). Immunostaining was performed in three independent biological experiments (*n* = 3) for cells treated with or without UK5099. For each condition, three representative images were acquired, and the mean gray value was measured three times per image using ImageJ software ImageJ (version 1.54 g, NIH, USA). Quantitative data represent the mean of these measurements. Immunostaining images were processed for brightness and contrast using Adobe Photoshop (ver.9.0, Adobe Inc., San Jose, CA, USA). All images were adjusted equally and no specific features were selectively enhanced or removed. Raw data are available in Supplementary Figure S3-1 to S3-3.

### Intracellular ATP measurement

Intracellular ATP concentrations in US2-KD cells were measured using the ATP Assay Kit-Luminescence (Dojindo Molecular Technologies, Kumamoto, Japan). US2-KD cells were washed with phosphate-buffered saline to remove the residual medium and metabolites. Subsequently, the cells were collected in serum-free DMEM and homogenized to release their intracellular components. Luminescence was measured using a microplate reader, and ATP concentrations were calculated based on a standard curve generated using ATP standards provided in the kit. ATP levels were measured at 48, 96, 144, and 192 h after differentiation induction. This process was performed in triplicate for each condition (with or without UK5099).

### Statistical analysis

Data analysis (including principal component analysis [PCA], heatmap analysis, and enrichment analysis) was conducted using MetaboAnalyst 6.0 (Wishart Research Group, Edmonton, AB, Canada) for advanced metabolomic profiling and statistical analyses. Basic data organization and descriptive statistics were performed using Microsoft^®^ Excel^®^ 2016 MSO (Microsoft Corporation, Redmond, WA, USA). Repeated analysis of variance (ANOVA) was employed to evaluate group effects (GEs), time effects (TEs), and interaction effects (IEs), whereas two-tailed paired t-tests were conducted to assess differences at specific time points. Data are presented as the mean ± standard deviation, with statistical significance defined as *p* < 0.05.

Sample size was determined based on an a priori power analysis using G*Power version 3.1.9.7 (Heinrich-Heine-Universität Düsseldorf, Düsseldorf, Germany), which indicated that *n* ≥ 20 per group would be required to achieve adequate statistical power. However, due to experimental and biological constraints, most experiments were conducted with *n* = 3 biological replicates per group, which is a commonly accepted standard for in vitro studies. For Western blotting, due to limited protein yield, experiments were performed in two independent biological replicates (*n* = 2), as described in the Methods section and figure legends.

## Results

**Fig. 1:**
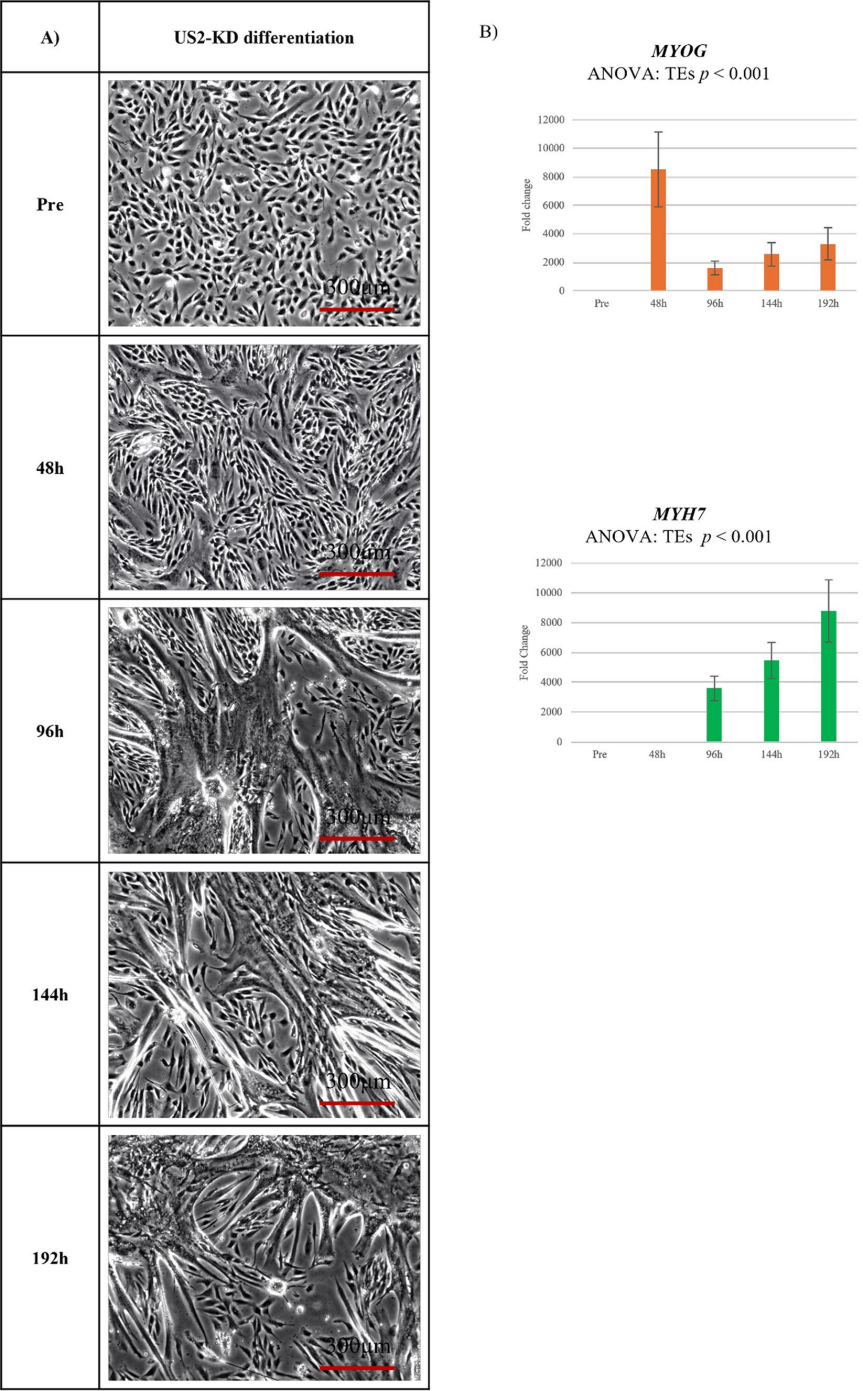
Differentiation of US2-KD myoblasts: Morphological changes and gene expression. (**A**) Phase-contrast images of US2-KD myoblasts during differentiation at 0 (Pre), 48, 96, 144, and 192 h. Scale bars = 300 μm. (**B**) Relative expression of *MYOG* (orange) and *MYH7* (green) mRNA by qRT-PCR. Data: mean ± SD; *n* = 3 biological replicates. Significant time effects (TEs): *p* < 0.001 (repeated measures ANOVA). h, hours.

Figure 1A shows that the differentiation process of US2-KD cells was observed. After 48 h, cell fusion progressed, and by 96 h, the formation of myotubes was evident. Subsequently, the maturation of myotubes continued. However, within the dish, the formation of muscle fibers proved challenging. After 144 h, the myotubes that had differentiated earlier began to detach, while those that differentiated later continued to mature. The raw data corresponding to Fig. 1A are provided in Supplementary Figure S4. Figure 1B revealed that *MYOG*, a muscle differentiation-promoting factor, peaked at 48 h, decreased at 96 h, and gradually increased thereafter (*p* < 0.001). *MYH7*, a muscle differentiation marker, showed significant elevation at 96 h (*p* < 0.001).

## Metabolic changes during hEUS myoblast differentiation

**Fig. 2:**
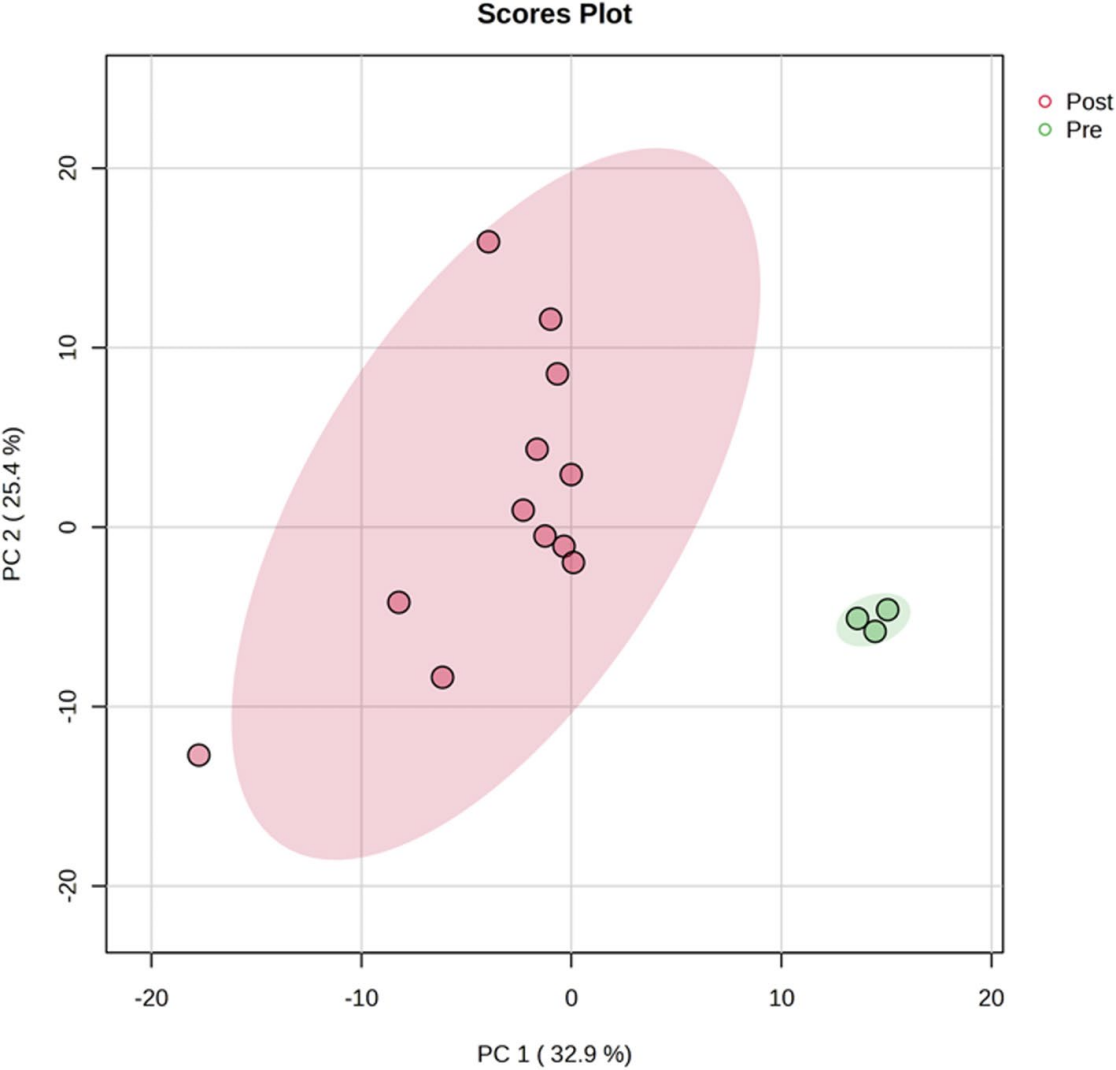
The results of principal component analysis (PCA) performed on metabolic profiles measured by GC-MS during the differentiation of human external urethral sphincter myoblasts. Pink points indicate samples before differentiation (Pre), while green points represent samples after differentiation (Post), including all four time points (48 h, 96 h, 144 h, and 192 h). Each point corresponds to an independent biological replicate (*n* = 3). GC-MS, gas chromatography-mass spectrometry. h, hours.

**Fig. 3:**
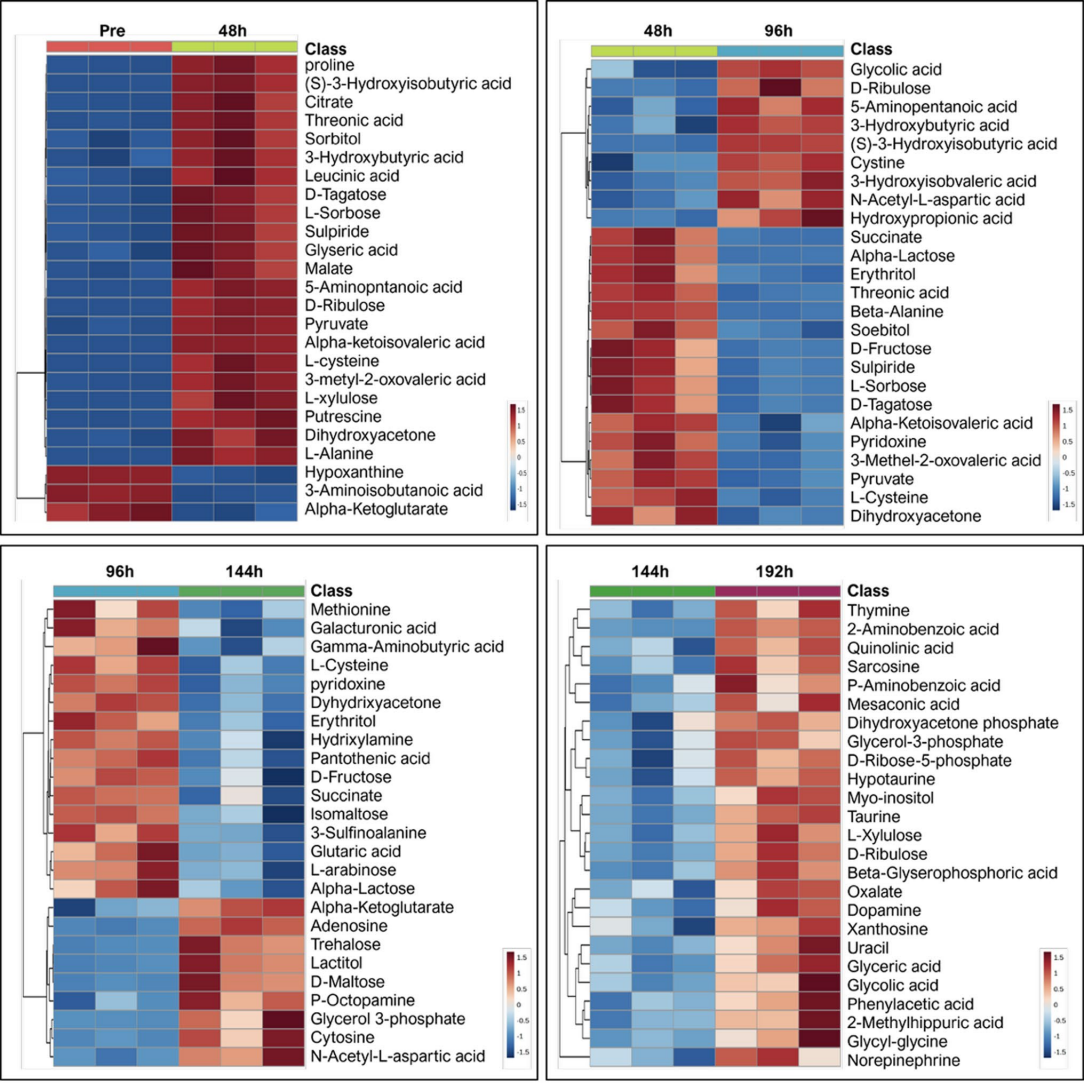
Dynamic metabolomic shifts during external urethral sphincter myoblast differentiation. The heatmap depicts the top 25 differentially abundant metabolites in the culture supernatant of differentiating external urethral sphincter myoblasts at 48-h intervals (0 [Pre]-48 h, 48–96 h, 96–144 h, and 144–192 h). Metabolomic profiling was performed in triplicate (*n* = 3) at each time point. h, hours.

**Fig. 4:**
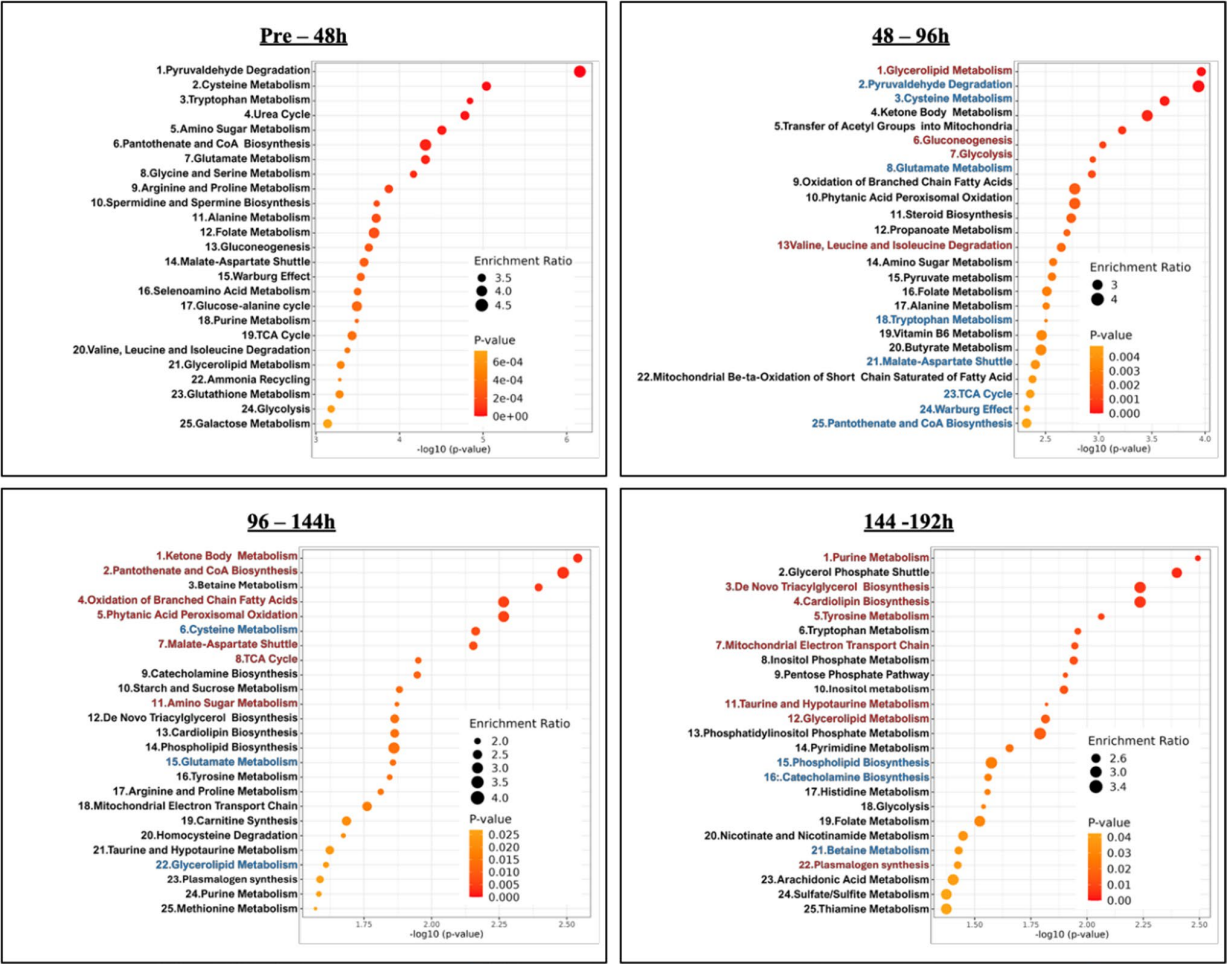
Overview of enriched metabolic pathways during differentiation of external urethral sphincter myoblasts. During the differentiation of external urethral sphincter myoblasts, culture supernatants were collected every 48 h and analyzed using GC–MS for metabolomic profiling. Based on the resulting data, pathway enrichment analysis was performed, and the top 25 statistically significant pathways for each time interval (pre–48 h, 48–96 h, 96–144 h, and 144–192 h) are presented.

**Fig. 5:**
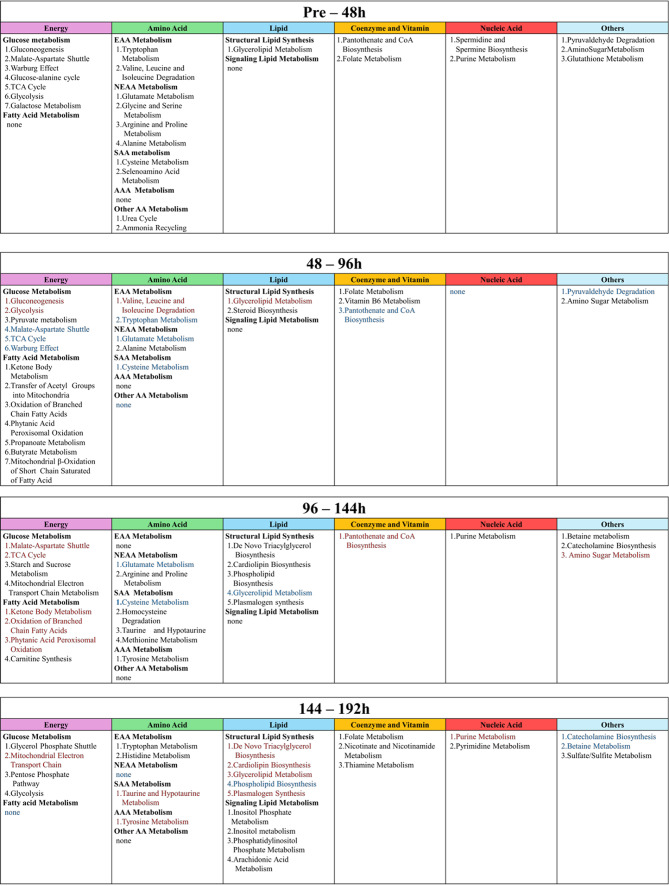
Dynamic classification of significant pathways during the differentiation of external urethral sphincter myoblasts. This figure presents the classification of the statistically significant metabolic pathways identified during the differentiation of external urethral sphincter myoblasts. Data were obtained from the culture supernatant collected every 48 h and analyzed using GC–MS. Pathways were grouped into six categories: energy, amino acid, lipid, coenzyme and vitamin, nucleic acid, and others. Black text indicates newly detected pathways, red text indicates pathways with increased rank compared with the previous interval, and blue text indicates pathways with decreased rank compared with the previous interval. TCA, tricarboxylic acid. h, hours. GC-MS, gas chromatography-mass spectrometry. EAA, essential amino acid. NEAA, non-essential amino acid. SAA, sulfur-containing amino acid. AA, amino acid.

We identified 208 metabolites in the culture supernatant during hEUS myoblast differentiation following a systematic filtering and selection workflow (Supplementary Fig.S5). PCA revealed a clear separation between pre-differentiation (0 h) and post-differentiation (48, 96, 144, and 192 h) samples along the first principal component (PC1), indicating distinct metabolic states before and after differentiation (Fig. 2). The heatmap (Fig. 3) depicts the top 25 metabolites and reveals distinct stage-specific patterns, demonstrating dynamic temporal shifts in metabolite abundance during the differentiation time course. Figure 4 lists the top 25 enriched pathways for each 48-hour interval, identified by comparing each time point to the immediately preceding time point (pre–48 h, 48–96 h, 96–144 h, and 144–192 h.). Multiple metabolic pathways showed statistically significant and time-dependent upregulation across these intervals. Details are as follows, focusing on energy metabolism. In the early stage (Pre–48 h), all pathways listed were activated with strong significance (*p* < 0.001), notably cysteine metabolism (enrichment ratio [ER]: 4.0), pantothenate and CoA biosynthesis (ER: 4.8), and several energy-related pathways such as gluconeogenesis (ER: 3.8), and the TCA cycle (ER: 4.0). During the mid-stage (48–96 h), glycerolipid (ER: 3.3, *p* < 0.001) and ketone body metabolism (ER: 4.4, *p* < 0.01) increased. In the late stage (96–144 h), ketone body metabolism (ER: 3.0, *p* < 0.001) and mitochondrial electron transport (ER: 3.2, *p* < 0.01) became more prominent. In the final stage (144–192 h), mitochondrial electron transport chain (ETC) activity peaked (ER: 2.6, *p* < 0.001), with enrichment of the glycerol-phosphate shuttle (ER: 3.3, *p* < 0.01), purine metabolism (ER: 2.4, *p* < 0.01), and pentose phosphate pathway (PPP, ER: 2.3, *p* < 0.02). A full list of pathways is provided in Supplementary Table S2-5. Figure 5 groups these pathways into six major metabolic categories: energy metabolism, lipid metabolism, amino acid metabolism, coenzyme and vitamin metabolism, nucleic acid metabolism, and other pathways.

## Effects of TCA cycle inhibition

Supplementary Figure S6 shows the time-course morphological changes of US2-KD myoblasts during differentiation without and with UK5099, revealing reduced myotube formation and altered cell morphology in treated cells compared to controls.

**Fig. 6:**
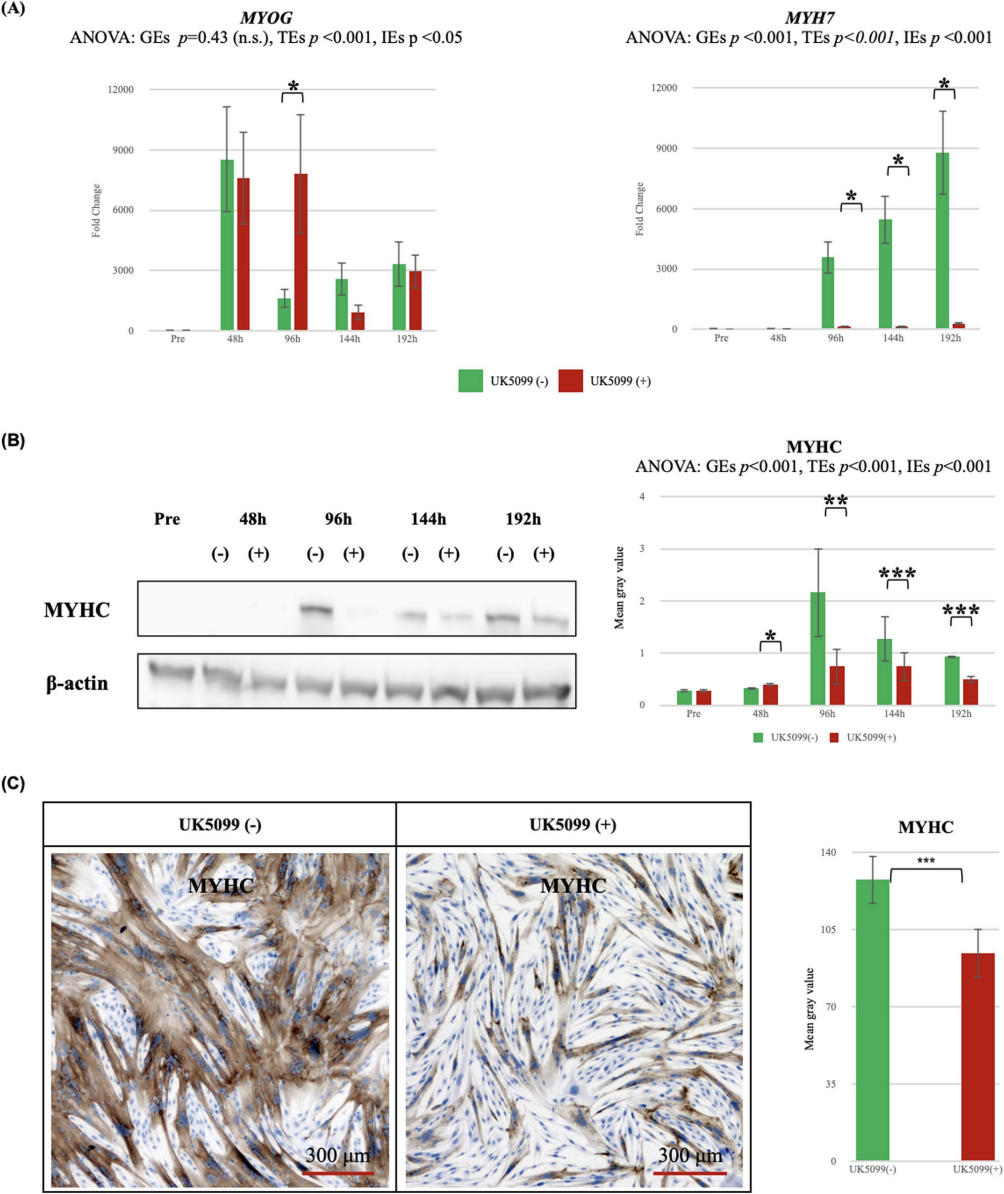
Inhibition of muscle differentiation by UK5099 in US2-KD myoblasts. (**A**) qRT-PCR analysis of *MYOG* and *MYH7* mRNA expression during differentiation (Pre, 48, 96, 144, 192 h) in US2-KD myoblasts treated with (red) or without (green) UK5099. *n* = 3. *p < 0.05, **p < 0.01. (**B**) Western blotting of MYHC protein at the same time points. β-actin was the loading control. (−): without UK5099; (+): with UK5099. *n* = 2; each blot quantified three times using ImageJ. Bar graph shows band intensities relative to β-actin (mean ± SD). Representative blots shown. Brightness and contrast were adjusted equally using Adobe Photoshop. Uncropped images are shown in Supplementary Figure S2-1 and S2-2. *p < 0.05, **p < 0.01, ***p < 0.001. (**C**) Phase-contrast and immunohistochemical images of MYHC at 96 h with or without UK5099. Representative images shown. Brightness and contrast were adjusted equally using Adobe Photoshop. Raw images are shown in Supplementary Figure S3-1 to S3-3. Quantification by ImageJ (mean ± SD, *n* = 3). ***p < 0.001. h, hours; GEs, group effects; TEs, time effects; IEs, interaction effects.

Treatment with the MPC inhibitor UK5099 showed no significant group effects on *MYOG* mRNA expression (GEs, *p* = 0.43 [n.s.]), while time effects (TEs, *p* < 0.001) and interaction effects (IEs, *p* < 0.05) were statistically significant (Fig. 6A). *MYH7* mRNA expression was significantly suppressed in UK5099-treated cells compared with that in untreated controls (GEs, *p* < 0.001; TEs *p* < 0.001; IEs *p* < 0.001; Fig. 6A). Western blotting confirmed the reduction in MYHC protein expression in the treated cells (Fig. 6B). At 48 h, MYHC expression in treated cells showed a slight increase, whereas untreated cells subsequently demonstrated a marked upregulation of MYHC expression from 96 h onward. Phase-contrast and immunohistochemical staining demonstrated diminished MYHC-positive myotube formation at 96 h in UK5099-treated cells (Fig. 6C). Quantitative analysis confirmed a significant reduction in MYHC immunostaining intensity in UK5099-treated cells compared to controls (*p* < 0.001).

**Fig. 7:**
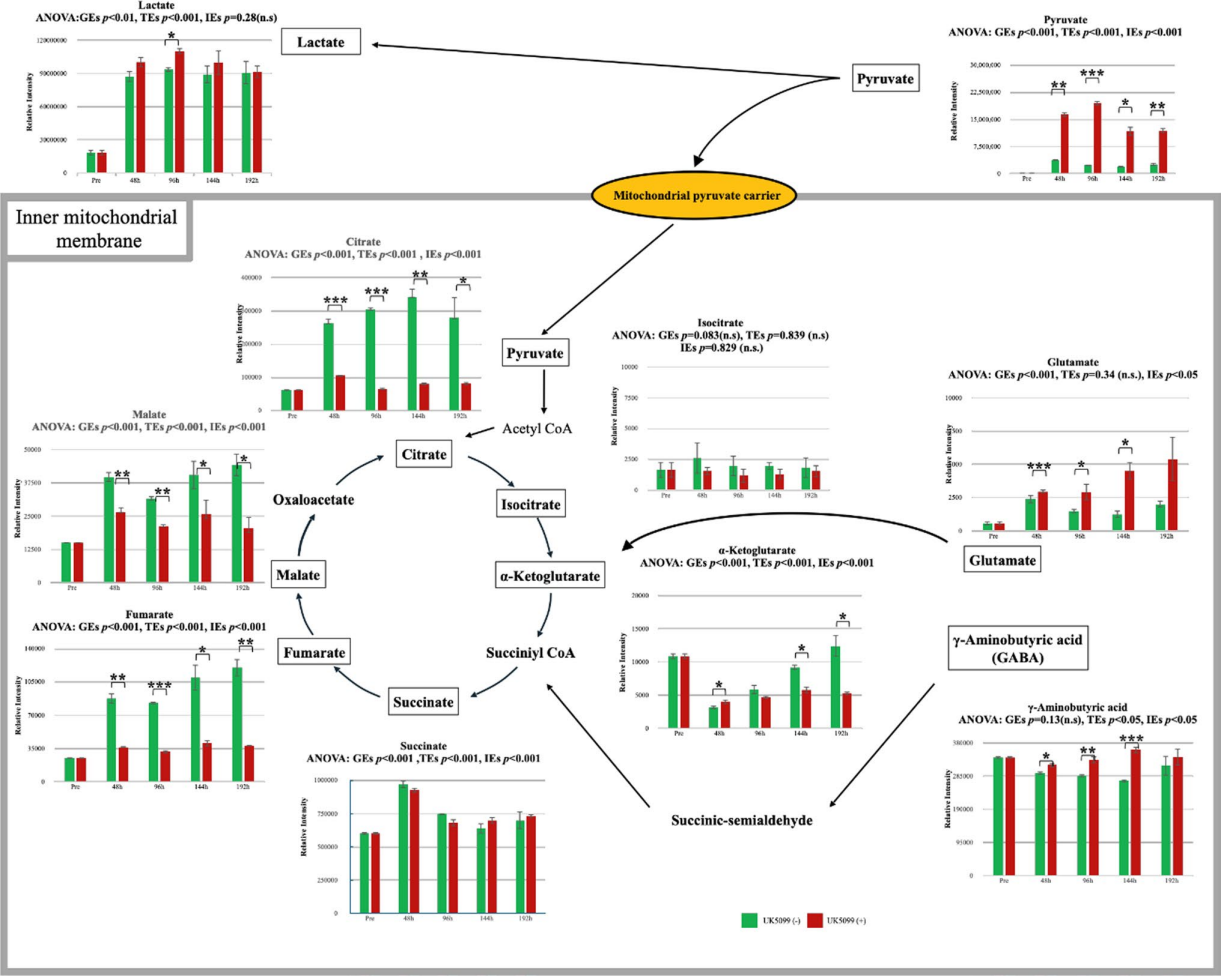
Effect of UK5099 on TCA cycle metabolite levels in culture supernatant during muscle differentiation. This figure presents the relative intensities of TCA cycle metabolites and related pathways in the culture supernatant of US2-KD cells during muscle differentiation, as analyzed by GC–MS. Green bars denote untreated US2-KD cells, whereas red bars indicate cells treated with UK5099. Statistical significance was determined using ANOVA, with GEs and TEs indicated. Significant differences are indicated by *p < 0.05, **p < 0.01, and ***p < 0.001. Error bars represent the standard deviation. (−): without UK5099 (+): with UK5099. TCA, tricarboxylic acid. h, hours. GC-MS, gas chromatography-mass spectrometry. GEs, group effects. TEs, time effects. IEs, interaction effects.

GC–MS analysis revealed significant metabolic alterations in UK5099-treated cells (Fig. 7), including the accumulation of pyruvate (GEs, *p* < 0.001; TEs, *p* < 0.001; IEs, *p* < 0.001) and lactate (GEs, *p* < 0.01; TEs, *p* < 0.001; IEs, *p* = 0.28 [n.s.]), indicating inhibition of mitochondrial pyruvate transport and subsequent disruption of the TCA cycle. The levels of TCA cycle intermediates, including citrate (GEs, *p* < 0.001; TEs, *p* < 0.001; IEs, *p* < 0.001), α-ketoglutarate (GEs, *p* < 0.001; TEs, *p* < 0.001; IEs, *p* < 0.001), succinate (GEs, *p* < 0.001; TEs, *p* < 0.001; IEs, *p* < 0.001), fumarate (GEs, *p* < 0.001; TEs, *p* < 0.001; IEs, *p* < 0.001), and malate (GEs, *p* < 0.001; TEs, *p* < 0.001; IEs, *p* < 0.001), were significantly reduced, whereas isocitrate levels remained unchanged (GEs, *p* = 0.83 [n.s.]; TEs, *p* = 0.84 [n.s.]; IEs, *p* = 0.83 [n.s.]). Glutamate was upregulated in UK5099-treated cells (GEs, *p* < 0.001; TEs, *p* = 0.34 [n.s.]; IEs, *p* < 0.05), while γ-aminobutyric acid (GABA; GEs, *p* = 0.13 [n.s.]; TEs, *p* < 0.05; IEs, *p* < 0.05) showed a significant interaction effect, indicating that its temporal change pattern differed between groups.

**Fig. 8:**
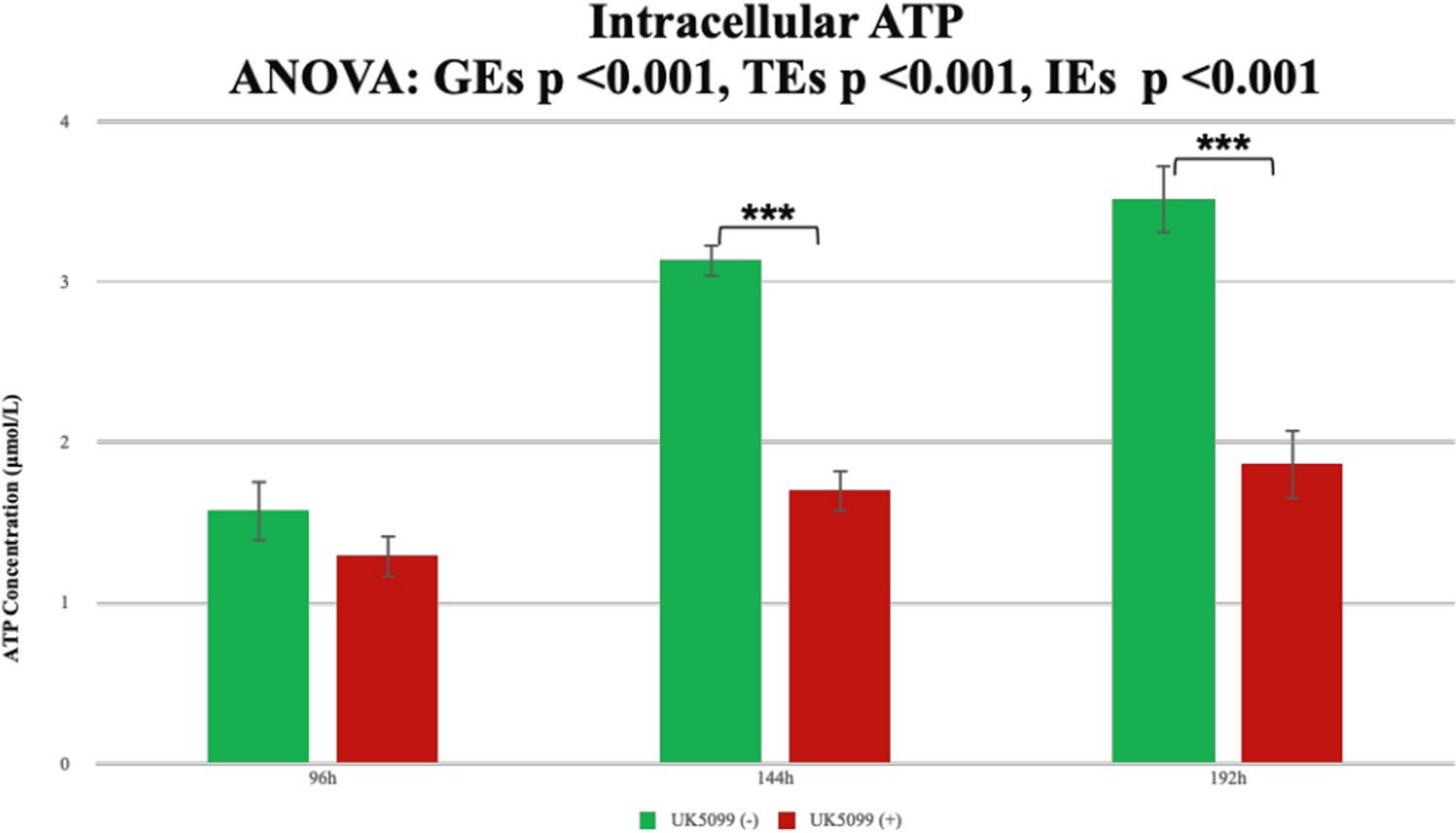
Intracellular ATP concentrations during muscle differentiation in US2-KD cells treated with UK5099. The intracellular ATP concentration (µmol/L) was measured in US2-KD cells during muscle differentiation at 96, 144, and 192 h. Green bars represent untreated cells, whereas red bars represent cells treated with UK5099. Statistical significance is indicated by *p < 0.05 and ***p < 0.001. Error bars represent the standard deviation. This process was performed in triplicate for each condition (with or without UK5099). h, hours. GEs, group effects. TEs, time effects. IEs, interaction effects.

Finally, to investigate the intracellular effects of TCA cycle inhibition, we measured ATP concentrations in cells treated with UK5099 (Fig. 8). Intracellular ATP concentrations were significantly reduced in UK5099-treated cells (GEs, *p* < 0.001; TEs, *p* < 0.001; IEs, *p* < 0.001).

## Discussion

This study aimed to elucidate the differentiation process of hEUS myoblasts through metabolic pathways and to investigate the effects of TCA cycle inhibition on hEUS myoblast differentiation.

The hEUS, like other skeletal muscles, is thought to maintain its satellite cells in a quiescent state and does not undergo continuous differentiation unless there is injury or specific stimulation. Activation requires stimuli such as secretome administration, pelvic floor muscle exercises, or neuromodulation therapy. In our preliminary experiments, with medium changes every 48 h as shown in Fig. 1, cell maintenance was enabled for over 100 days. However, repeated cycles of myotube formation and detachment (turnover) occurred, and after around 80 days, fibroblasts predominated and myotube formation capacity was permanently lost.

### Metabolomics of hEUS myoblast differentiation

Integration of Figs. 2, 3 and 4 reveals not only the activation of specific metabolic pathways but also a dynamic reprioritization of pathway utilization during hEUS myoblast differentiation. While several pathways—including glycolysis, cysteine metabolism, and ketone body metabolism—were transiently upregulated or maintained high activity at specific stages, others showed a relative decrease in prioritization over time. For example, glycolysis and the Warburg effect were prominent in the early stage but diminished as cells shifted toward mitochondrial oxidative phosphorylation and ketone body metabolism in later stages. This temporal change does not necessarily indicate a simple downregulation or suppression of these pathways; rather, it reflects metabolic reprogramming, in which the relative contribution of each pathway is adjusted according to the cellular demands at each differentiation stage.

Amidst this dynamic metabolic reprogramming, the present study focused particularly on changes in energy metabolism. Notably, pantothenate CoA biosynthesis remained consistently active as a coenzyme source for the TCA cycle throughout all stages of differentiation, thereby supporting mitochondrial energy production stability. Furthermore, ATP efficiency increased stepwise at each stage, indicating a progressive optimization of energy metabolism as differentiation proceeded.

In the early stage, cysteine metabolism plays a significant role in maintaining cellular redox balance by providing antioxidant defense through glutathione synthesis. Tryptophan metabolism also contributes to NAD + synthesis and cell signaling, which are necessary for muscle differentiation. Additionally, the arginine-proline metabolic pathway was significantly activated, with proline synthesis contributing to membrane formation and energy preparation in coordination with glutamate metabolic pathways. These have been reported to be important functions in the early stages of differentiation in skeletal muscle as well^17,18^.

In the mid-stage, elevated glycolic acid (GA) levels suggested its involvement in energy supply pathways and mitochondrial function, potentially entering the TCA cycle via the oxalate pathway or enhancing mitochondrial efficiency. While GA’s association with muscle cells has been limited, this study highlights its potential role in supporting differentiation^19,20^. Concurrently, activation of ketone body metabolism significantly enhanced energy production. The concentration of 3-hydroxybutyrate (3-HB), a major ketone body metabolite, was elevated. This metabolic shift reflects the cell’s adaptation to more efficient energy utilization during differentiation, while glycerolipid metabolism supported membrane synthesis necessary for myotube formation.

In the late stage, ketone body metabolism became more prominent, further enhancing energy production efficiency^21^. For the first time during the differentiation process, mitochondrial electron transport chain (ETC) activity was induced, enabling efficient ATP production through oxidative phosphorylation. Elevated adenosine and oxaloacetate levels supported this transition to efficient energy metabolism, while betaine metabolism contributed antioxidant functions by promoting homocysteine remethylation to methionine^22,23^. These shifts indicate that cells optimize energy metabolism while constructing themselves.

In the final stage, the ETC reached peak activity, and the glycerol-phosphate shuttle facilitated the conversion of NADH to FADH₂, enabling additional electron transport and further increasing ATP efficiency. Nucleic acid synthesis pathways and PPP provided NADPH for redox regulation and nucleotide precursors for transcriptional activity essential for cellular maturation^24^. Elevated sarcosine levels suggested autophagy induction, promoting cellular homeostasis during this period^25^.

In summary, hEUS myoblast differentiation involves distinct metabolic phases: early resource accumulation with glycolysis-driven ATP production, mid-stage mitochondrial activation with ketone body metabolism, late-stage efficient oxidative phosphorylation, and final-stage maximal ATP efficiency, alongside nucleic acid synthesis and autophagy regulation.

### Comprehensive metabolic pathway analysis

This comprehensive analysis illustrated the utility of metabolomic profiling in uncovering novel functions of metabolites that initially appeared unrelated to hEUS myoblast differentiation. For example, changes in metabolomes in culture supernatant can help infer intracellular changes when metabolic pathways are well understood. Similar to clinical blood tests, in which biomarkers such as C-reactive protein or creatinine indicate systemic conditions, analyzing culture supernatants in combination with other omics analyses enables the inference of intracellular events from extracellular information. This approach identifies unknown pathways but also highlights previously underappreciated roles of metabolites and processes such as GA or PPP activation during muscle differentiation^19,20,22,26^.

### Changes in hEUS myoblast differentiation by TCA cycle inhibition

Comprehensive elucidation of metabolic pathways revealed that metabolites can be categorized into six major pathways (Fig. 5). Among these, the TCA cycle is of particular interest due to its central role not only in energy metabolism but also in amino acid, lipid, coenzyme vitamin, and nucleic acid metabolism. In terms of lipid metabolism, fatty acids enter the TCA cycle and contribute to energy production. Amino acid metabolism involves intermediates such as pyruvate and α-ketoglutarate, which flow into the TCA cycle to support continuous metabolic flux. Many enzymes within the TCA cycle require vitamin-derived compounds as coenzymes, highlighting the importance of coenzyme vitamin metabolism. Furthermore, the purine nucleotide cycle and pyrimidine metabolism, which are key pathways in nucleic acid metabolism, generate intermediates like fumarate and succinyl-CoA that connect directly to the TCA cycle.

Given this centrality, it is plausible that TCA cycle inhibition could impair cellular functions, including differentiation. However, there is currently no experimental evidence elucidating how such inhibition specifically affects differentiation in the human external urethral sphincter.

The second objective of this study was to investigate the impact of TCA cycle inhibition on hEUS myoblast differentiation. We employed UK5099, a potent MPC inhibitor, for this purpose.

UK5099 treatment significantly suppressed differentiation in US2-KD cells (Fig. 6). Although MPC inhibition has been reported to inhibit skeletal muscle differentiation in C2C12 cells^27^, this study provides the first evidence of this effect in hEUS myoblasts.

Notably, while MYOG expression persisted after UK5099 treatment, differentiation was arrested, suggesting myogenin-independent regulation of hEUS myoblast differentiation (Fig. 6A). We are considering the possibility that this dissociation between myogenin expression and differentiation arrest may be due to downregulation of AKT phosphorylation caused by reduced ATP levels, as suggested by Supplementary Figure S7. AKT signaling is known to regulate myogenic differentiation through pathways distinct from myogenin transcriptional control, which could potentially explain the observed differentiation block despite sustained myogenin expression^28,29^. As shown in Fig. 6B, at 48 h, MYHC expression in UK5099-treated cells showed a slight increase, possibly reflecting compensatory mechanisms such as glutamate metabolism. However, as differentiation progressed, these compensatory pathways appeared insufficient to meet increasing ATP demands, resulting in marked suppression of myoblast differentiation at later time points (Fig. 6B and C). Intriguingly, preliminary data suggested enhanced differentiation at low UK5099 concentrations, implying context-dependent effects of compensatory metabolism (e.g., glutamate metabolism). This implies that when compensatory mechanisms sufficiently counterbalance MPC inhibition, they may promote hEUS myoblast differentiation.

Following the discussion of compensatory glutamate metabolism in response to TCA cycle inhibition, we analyzed TCA cycle-related metabolites in the culture supernatant to elucidate the mechanism underlying the suppression of differentiation (Fig. 7). UK5099 inhibited mitochondrial pyruvate transport, profoundly suppressing metabolites including citrate, α-ketoglutarate, fumarate, and malate^16,27^. This triggered compensatory activation of glutamate and GABA-related pathways. Although the culture medium GABA is primarily FBS-derived, our data indicate that muscle cells utilize the GABA shunt to replenish succinate. Succinate also exhibited compensatory elevation, but failed to restore downstream intermediates (fumarate and malate), demonstrating the functional irreplaceability of the TCA cycle. This finding is consistent with the intracellular gene and protein expression profiles shown in Fig. 6.

To investigate whether TCA cycle inhibition in the culture supernatant leads to insufficient intracellular energy for hEUS myoblast differentiation, intracellular ATP levels were measured at 96 h 100results unequivocally demonstrate that TCA cycle inhibition halts energy production, depletes intracellular ATP, and suppresses hEUS myoblast differentiation. The downstream metabolic consequences of ATP reduction are partially detailed in Supplementary Figure S7. These findings suggest potential myogenin-independent pathways in hEUS myoblast differentiation suppression and indicate that culture supernatant analysis may provide insights into intracellular states.

### Clinical implications

This study elucidated the importance of the TCA cycle in hEUS myoblast differentiation through metabolomic analysis, and observed that TCA cycle inhibition disrupts energy metabolism, thereby suppressing the differentiation process. These findings suggest that metabolic vulnerabilities associated with aging contribute to the pathogenesis of SUI, positioning this study within the framework of translational research at the T1 stage. Furthermore, existing drugs such as pyruvate (TCA substrate)^30^, metformin (AMPK modulator)^31^, and imeglimin (mitochondrial complex I/III enhancer)^32^ have been reported to improve mitochondrial function and promote myoblast differentiation in skeletal muscle, highlighting their potential applicability in the treatment of SUI.

Current SUI treatment involves localized approaches such as PRP injections or stem cell transplantation around hEUS tissue and have shown therapeutic benefits. However, their long-term efficacy remains limited, potentially because of insufficient metabolic support in aging tissues^4–9^. The findings of this study indicate that combining oral TCA cycle–targeted drugs (e.g., imeglimin, pyruvate, and metformin) with PRP therapy could represent a novel therapeutic strategy. This integrated approach could achieve synergistic effects by combining structural repair facilitated by PRP with the metabolic support provided by TCA cycle activators, enabling sustained therapeutic outcomes. Moreover, the results of this study provide a foundation for the progression to T2 research (clinical trials) aimed at evaluating the efficacy of age-related SUI treatments.

### Limitations

Although this study demonstrated the relationship between TCA cycle activity and hEUS myoblast differentiation using an in vitro experimental system, several limitations must be considered. First, the study utilized a cell line derived from a single male patient, which may limit the generalizability of the findings. Moreover, the complexity of the in vivo environment cannot be fully replicated in this experimental setting, potentially affecting the applicability of the results to living organisms. It should also be noted that UK5099, while primarily an MPC inhibitor, may exert off-target effects, including downregulation of STAT3, which could confound interpretations^27^. The absence of positive controls, such as TCA cycle activators like pyruvate, represents another limitation in validating the specificity of the observed effects. Additionally, the metabolic profiling in this study relied exclusively on GC-MS analysis, which does not directly measure cellular metabolic flux dynamics; complementary assays such as Seahorse XF Analyzer measurements of oxygen consumption rate and extracellular acidification rate would provide valuable real-time insights. Attention must also be paid to the detection limits inherent to the GC-MS system used. Finally, the small sample size may have resulted in insufficient statistical power, limiting the detection of subtle effects and reproducibility. To address these issues, future studies should incorporate animal models and utilize comprehensive multiomics approaches.

## Conclusions

Our metabolic analysis identified the pathways involved in hEUS myoblast differentiation based on metabolite profiles and revealed a strong association between the differentiation process and the TCA cycle. The results of culture supernatant analysis were consistent with the observed phenotypes, demonstrating its utility as a simplified tool for evaluating cellular states. The finding that TCA cycle inhibition alters the metabolic pathways of hEUS myoblasts provides new therapeutic opportunities for SUI in the future.

Black text indicates newly detected pathways, red text indicates pathways with increased rank compared with the previous time interval, and blue text indicates pathways with decreased rank compared with the previous time interval. Three independent biological replicates were used for metabolomic analysis (*n* = 3). The full results of pathway enrichment analysis, including all tested pathways, number of hits, and p values, are provided in Supplementary Table S2-5. TCA, tricarboxylic acid. h, hours. GC-MS, gas chromatography-mass spectrometry.

## Supplementary Information

Below is the link to the electronic supplementary material.

Supplementary Material 1

Supplementary Material 2

Supplementary Material 3

Supplementary Material 4

Supplementary Material 5

Supplementary Material 6

Supplementary Material 7

Supplementary Material 8

Supplementary Material 9

Supplementary Material 10

Supplementary Material 11

Supplementary Material 12

Supplementary Material 13

Supplementary Material 14

Supplementary Material 15

## Data Availability

The datasets generated and/or analyzed during the current study are available from the corresponding author on reasonable request.
