# Supplementary figures and images for: Metabolic dynamics of human external urethral sphincter myoblast differentiation and the effects of tricarboxylic acid cycle inhibition
Source: Sci Rep. 2025 Aug 7;15:28987. doi: 10.1038/s41598-025-13764-z (PMC12331984; doi:10.1038/s41598-025-13764-z)

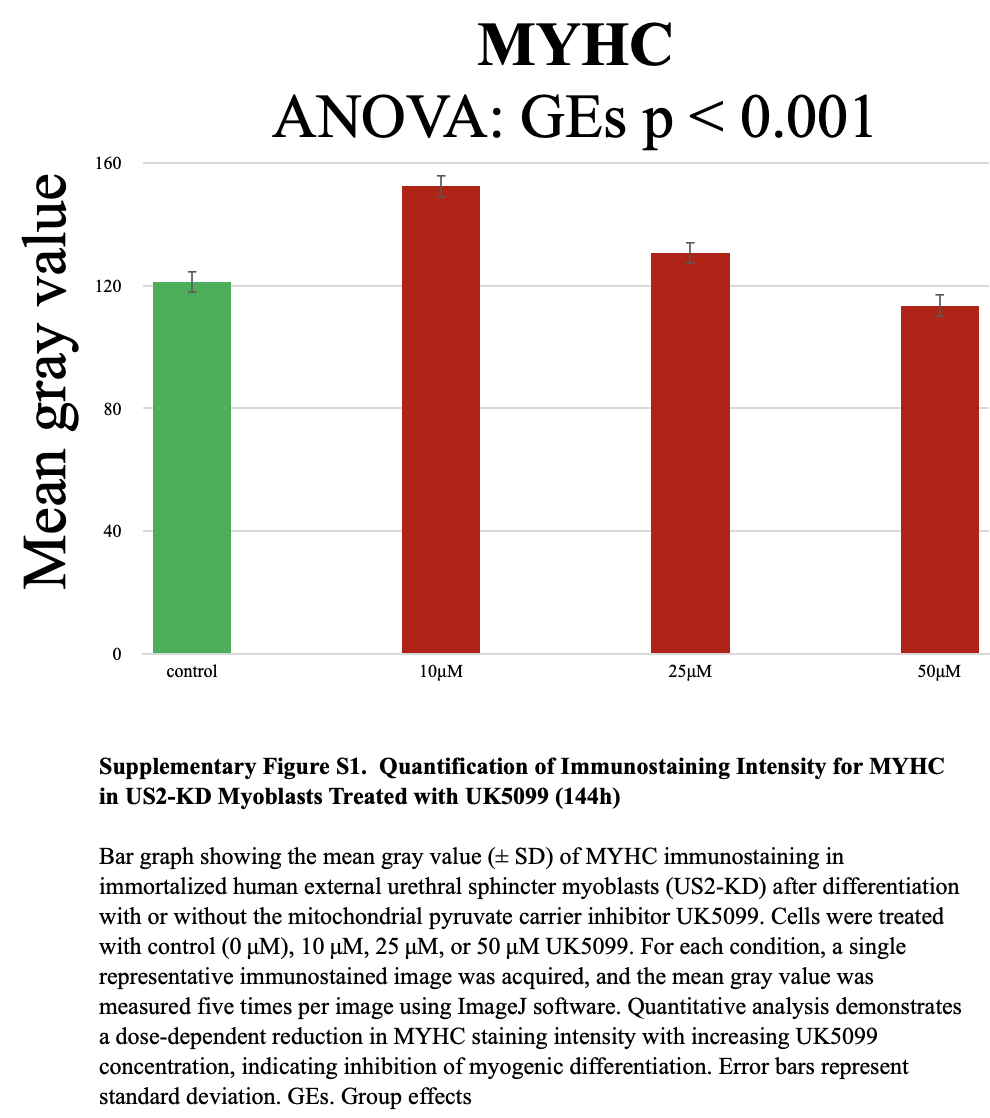

Supplement: Supplementary file 1 — Supplementary Material 1 [file 41598_2025_13764_MOESM1_ESM.tiff]

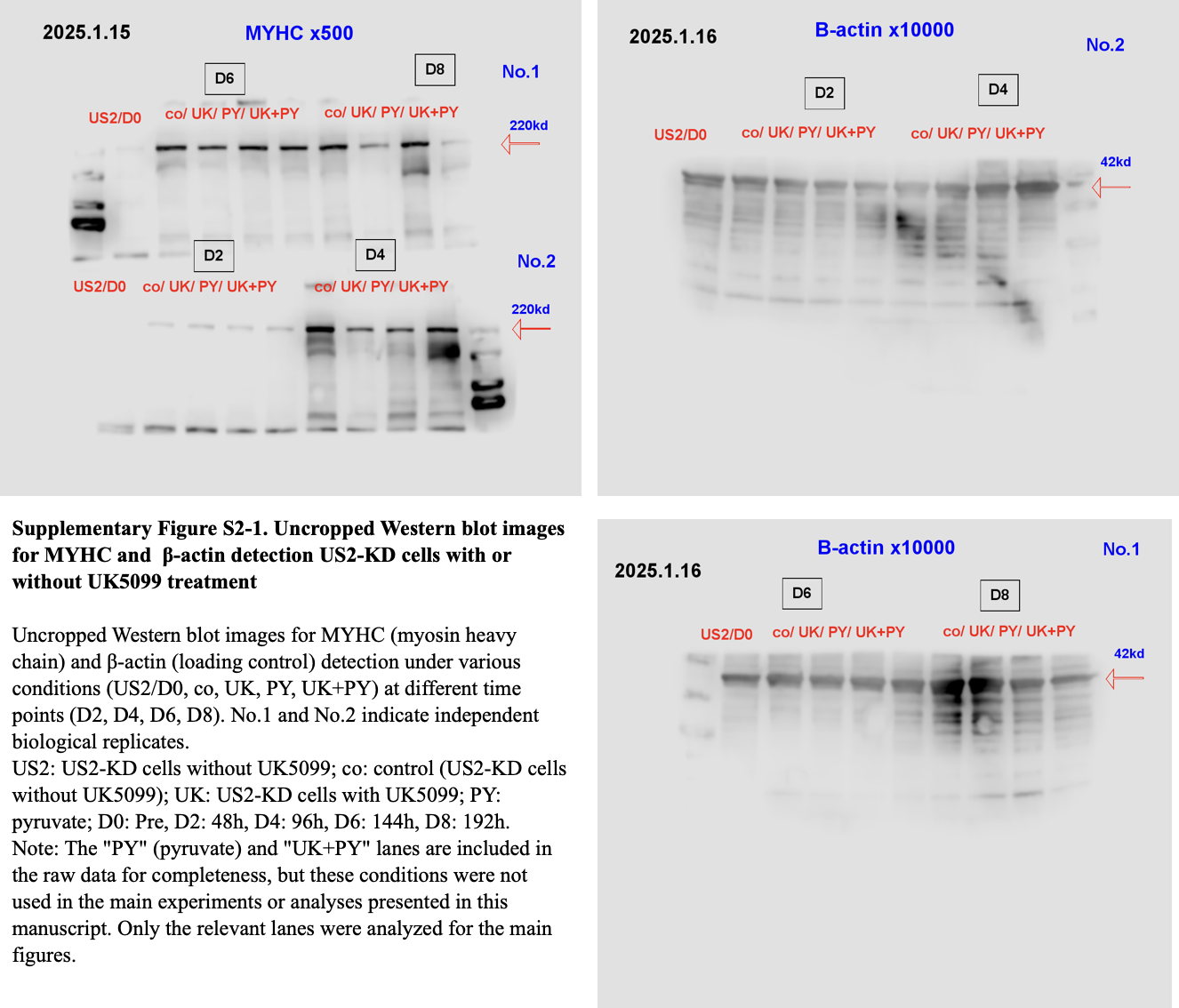

Supplement: Supplementary file 2 — Supplementary Material 2 [file 41598_2025_13764_MOESM2_ESM.tiff]

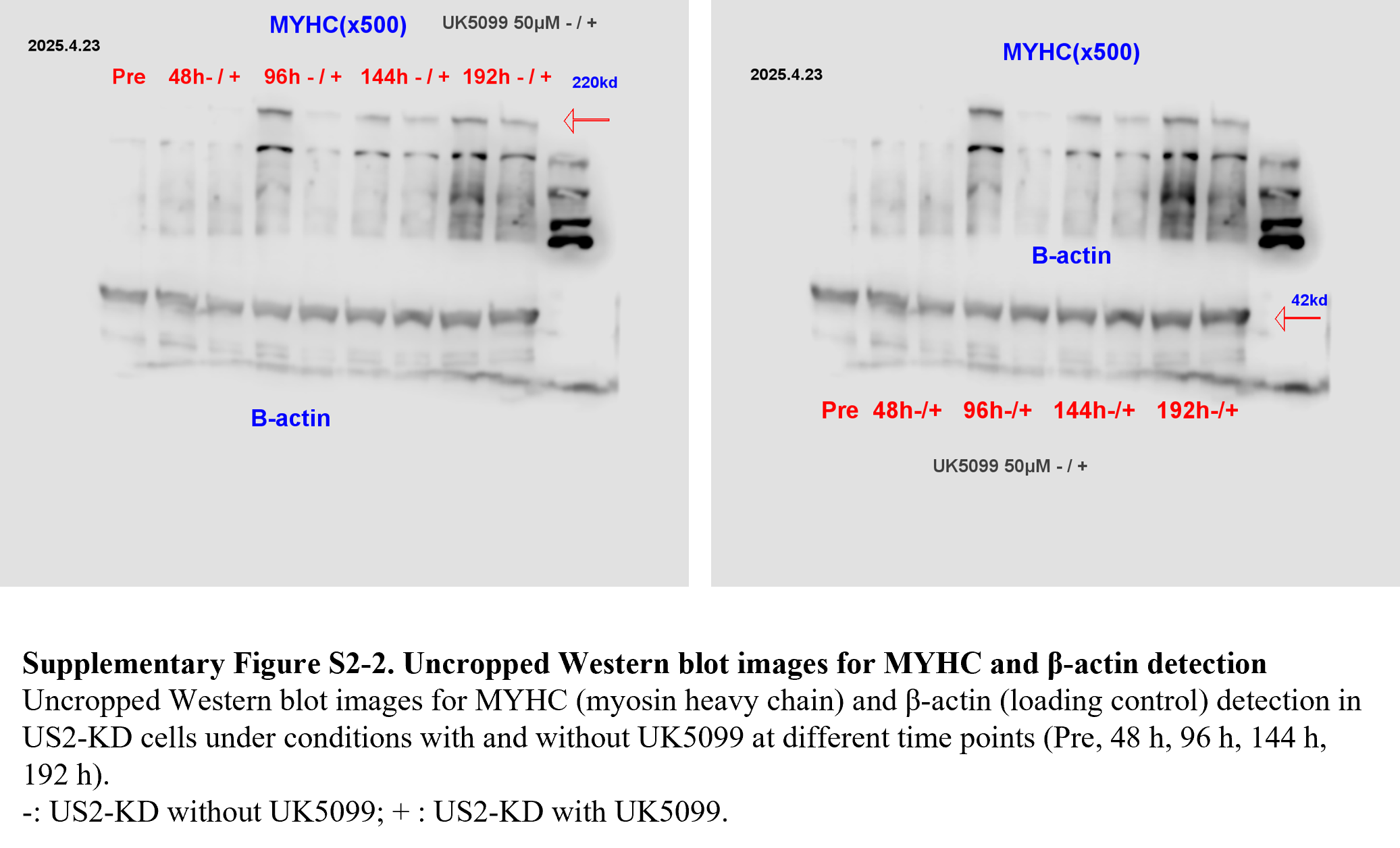

Supplement: Supplementary file 3 — Supplementary Material 3 [file 41598_2025_13764_MOESM3_ESM.tiff]

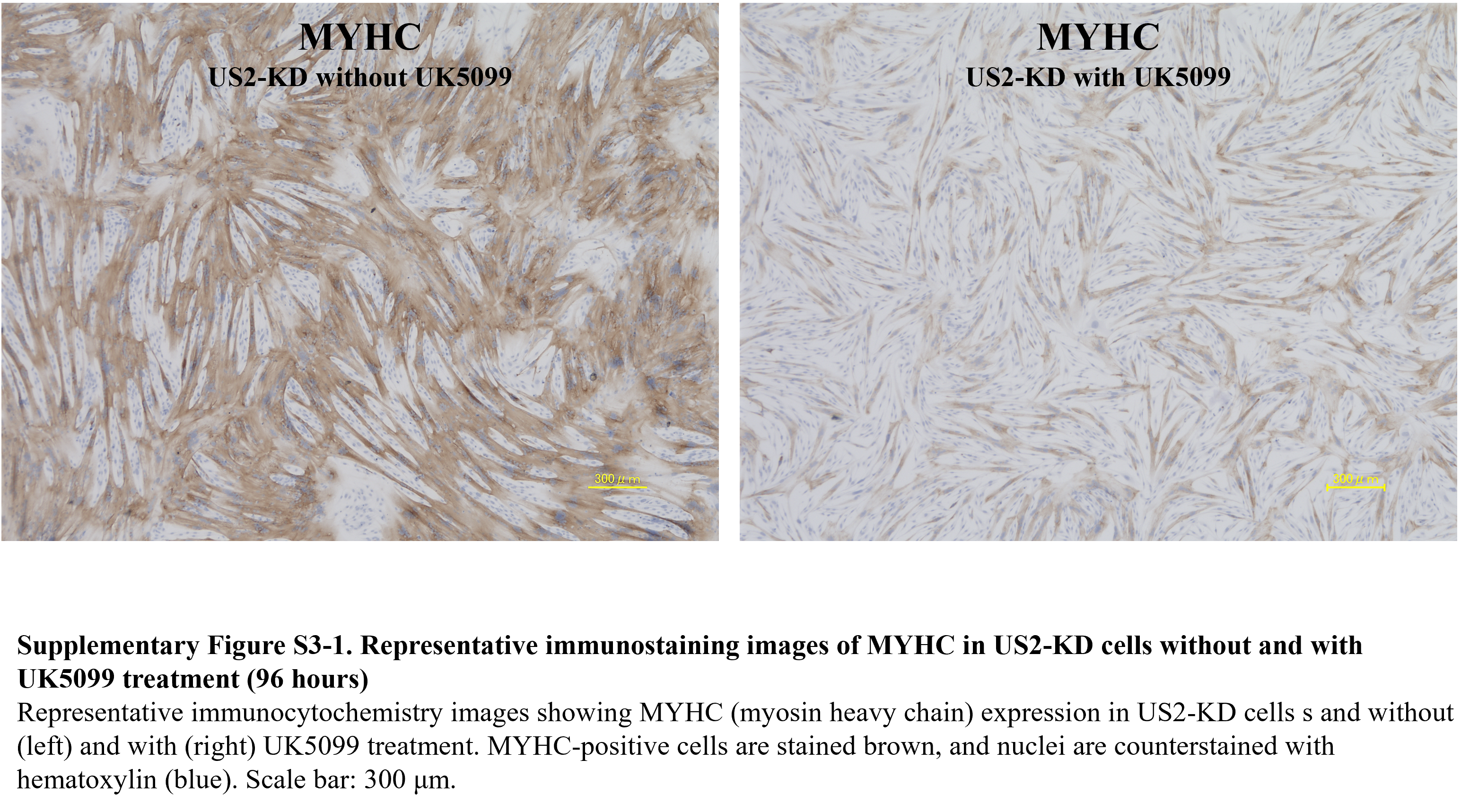

Supplement: Supplementary file 4 — Supplementary Material 4 [file 41598_2025_13764_MOESM4_ESM.tiff]

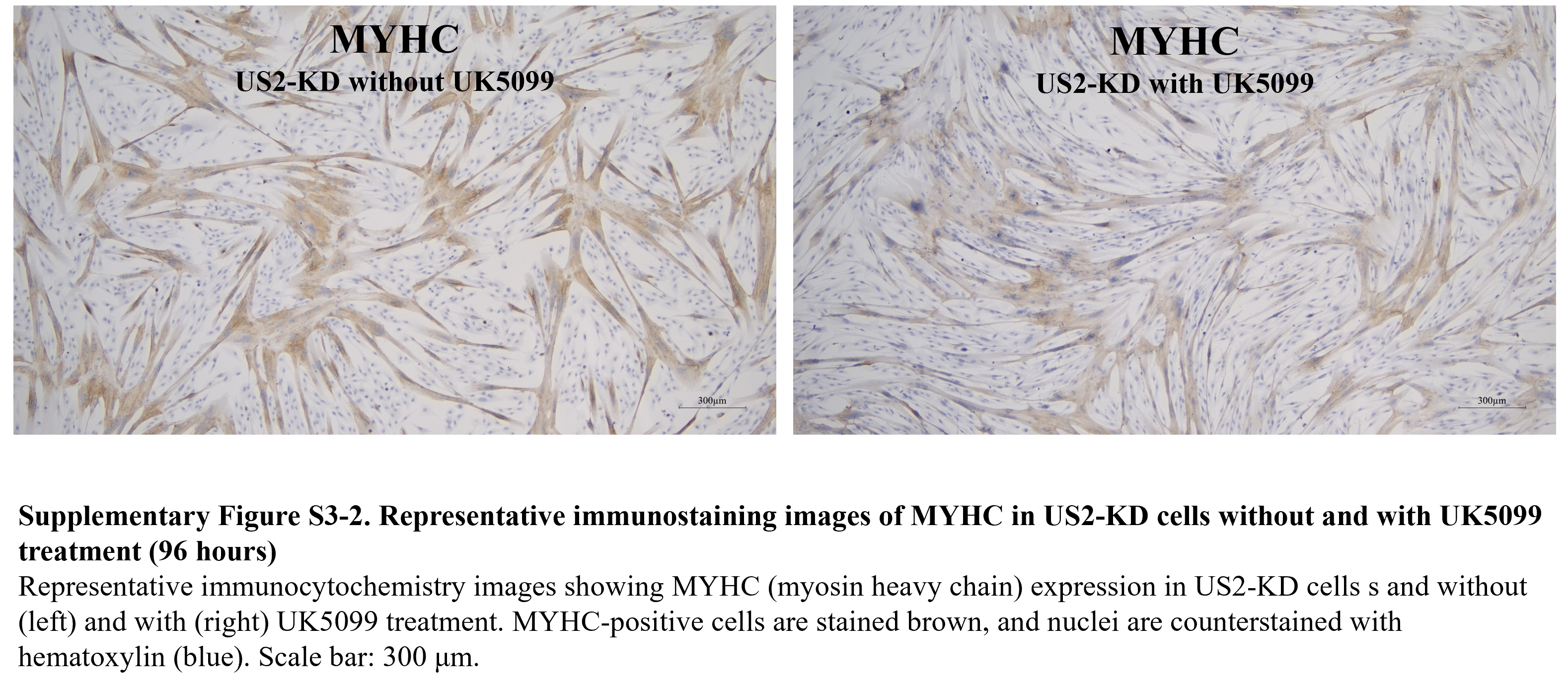

Supplement: Supplementary file 5 — Supplementary Material 5 [file 41598_2025_13764_MOESM5_ESM.tiff]

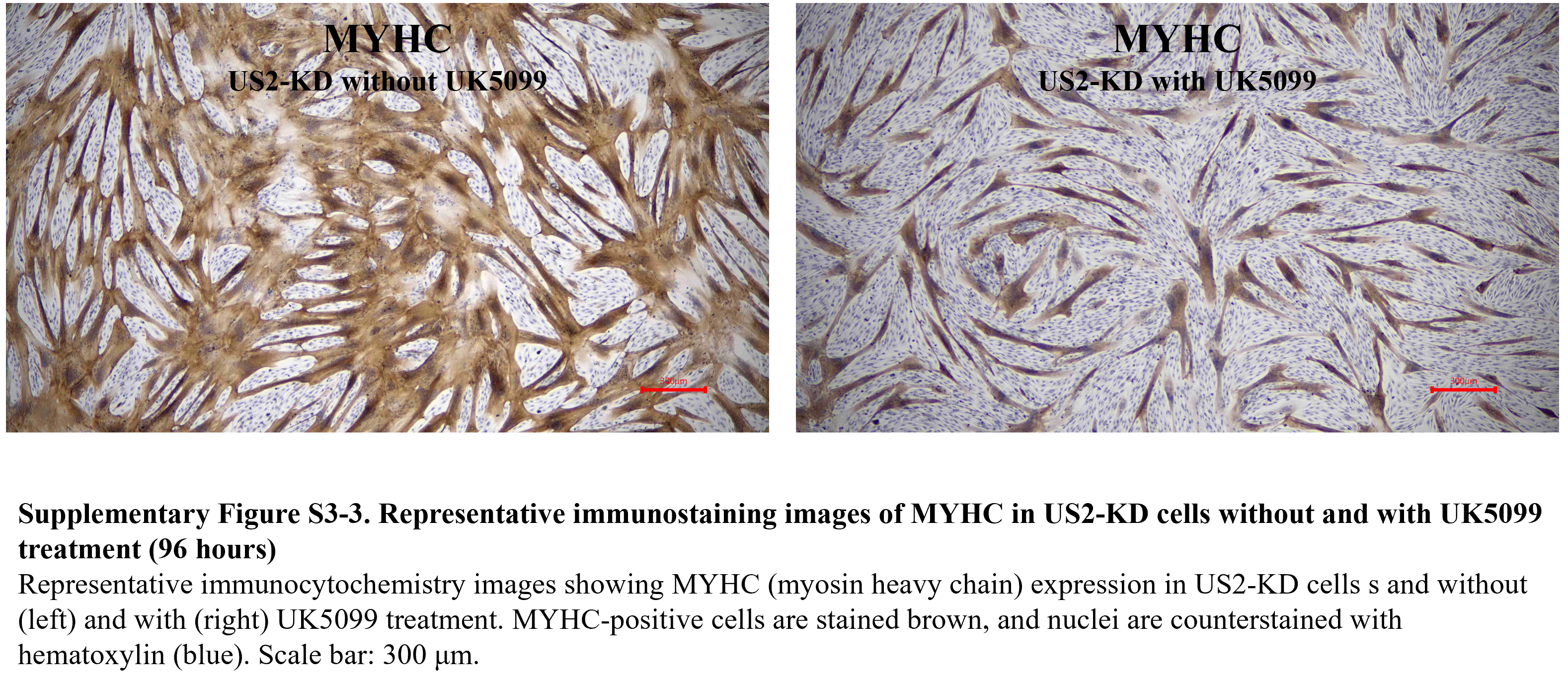

Supplement: Supplementary file 6 — Supplementary Material 6 [file 41598_2025_13764_MOESM6_ESM.tiff]

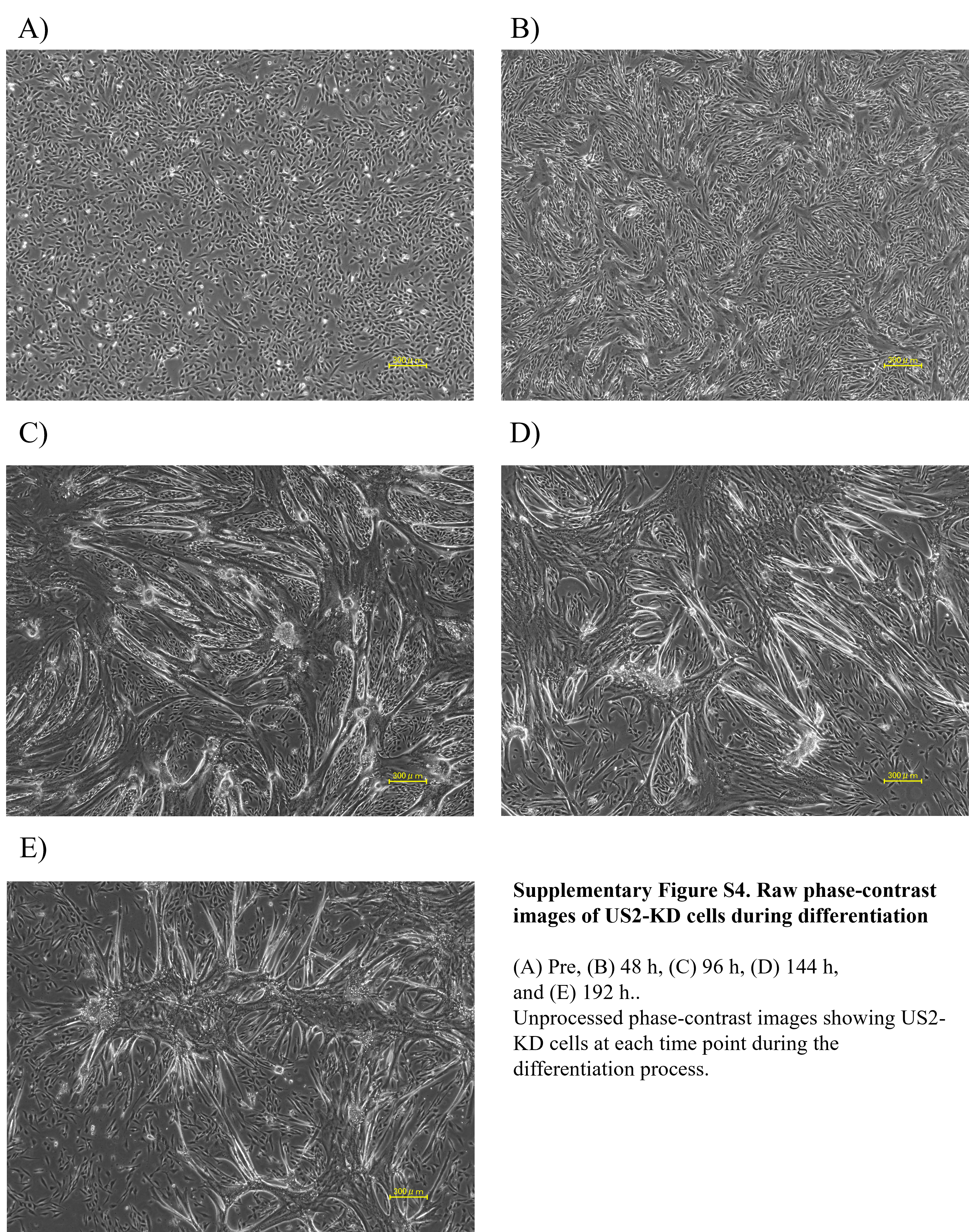

Supplement: Supplementary file 7 — Supplementary Material 7 [file 41598_2025_13764_MOESM7_ESM.tiff]

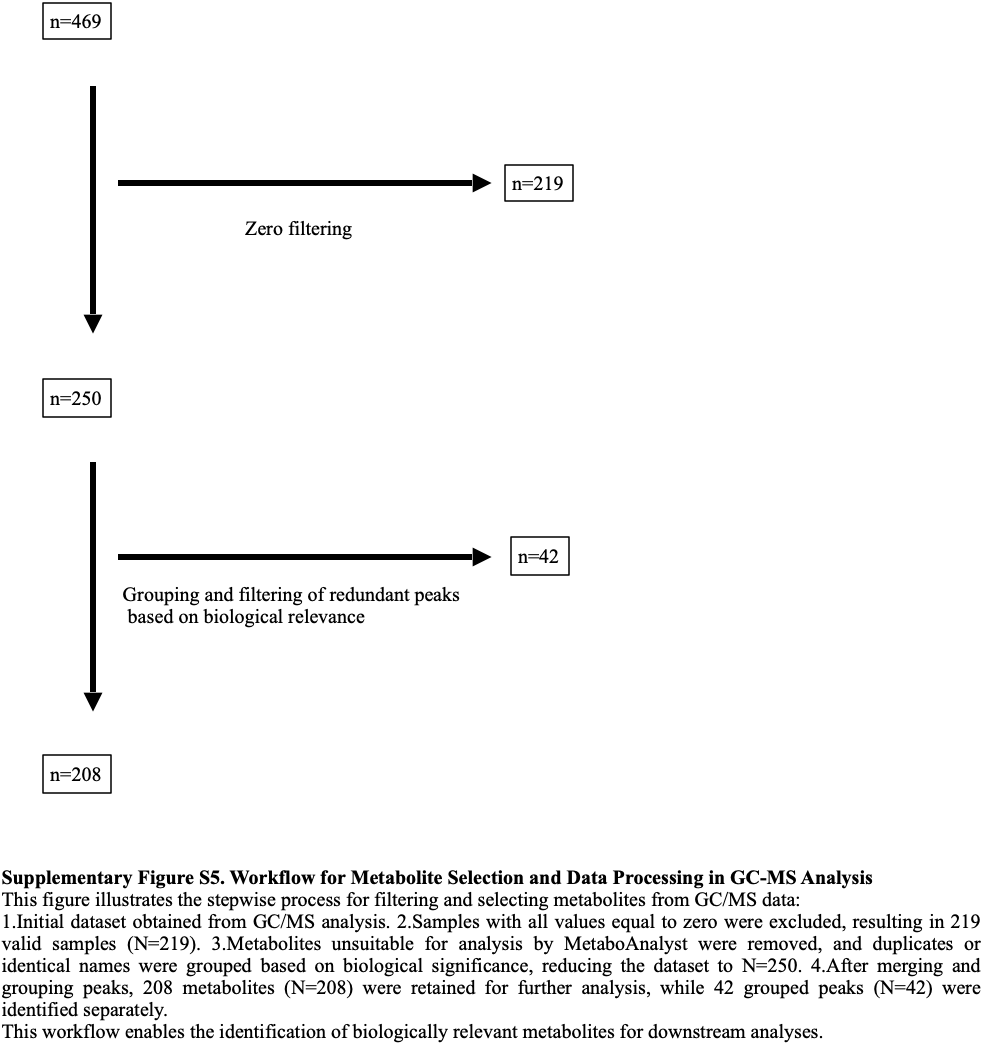

Supplement: Supplementary file 8 — Supplementary Material 8 [file 41598_2025_13764_MOESM8_ESM.tiff]

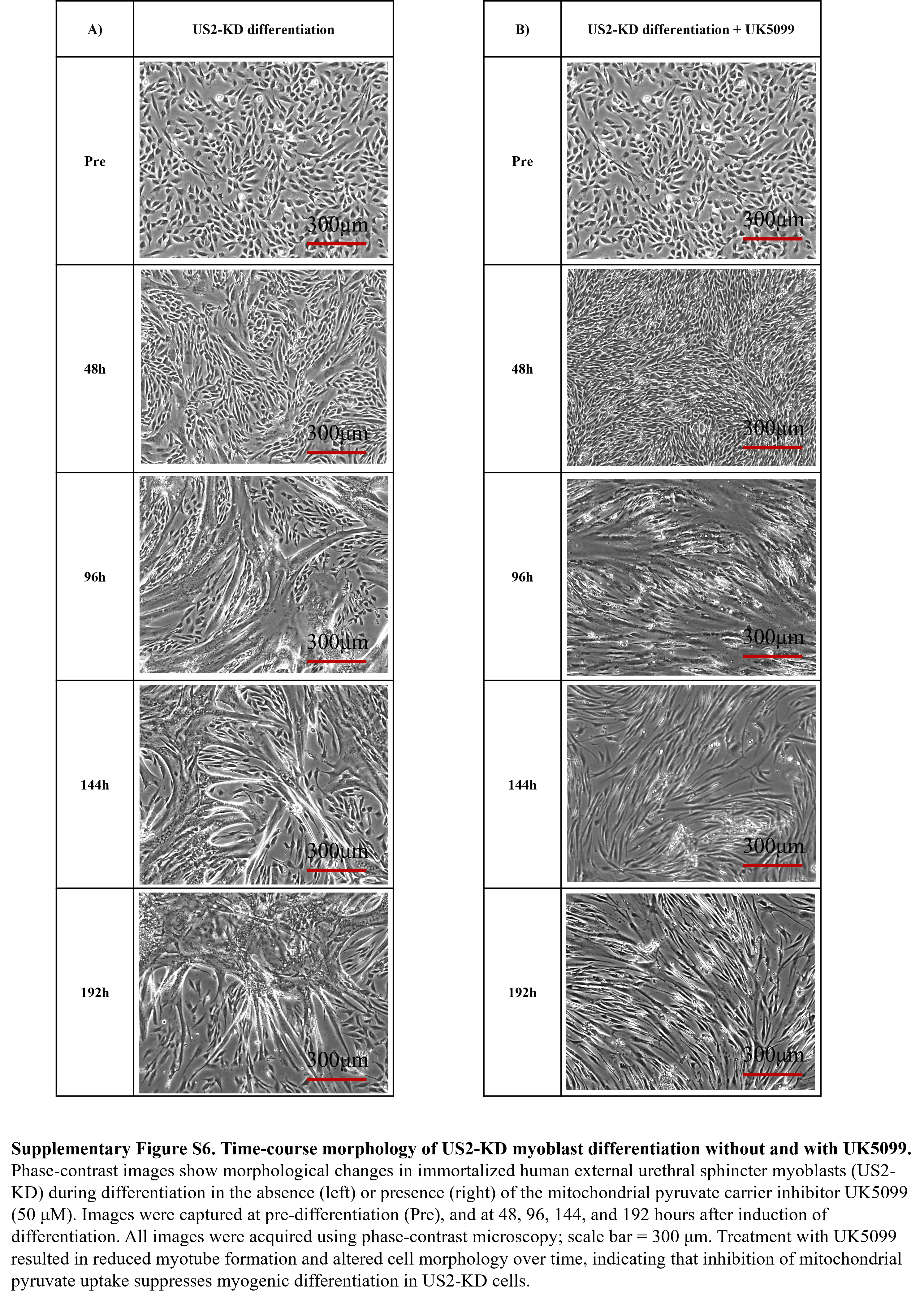

Supplement: Supplementary file 9 — Supplementary Material 9 [file 41598_2025_13764_MOESM9_ESM.tiff]

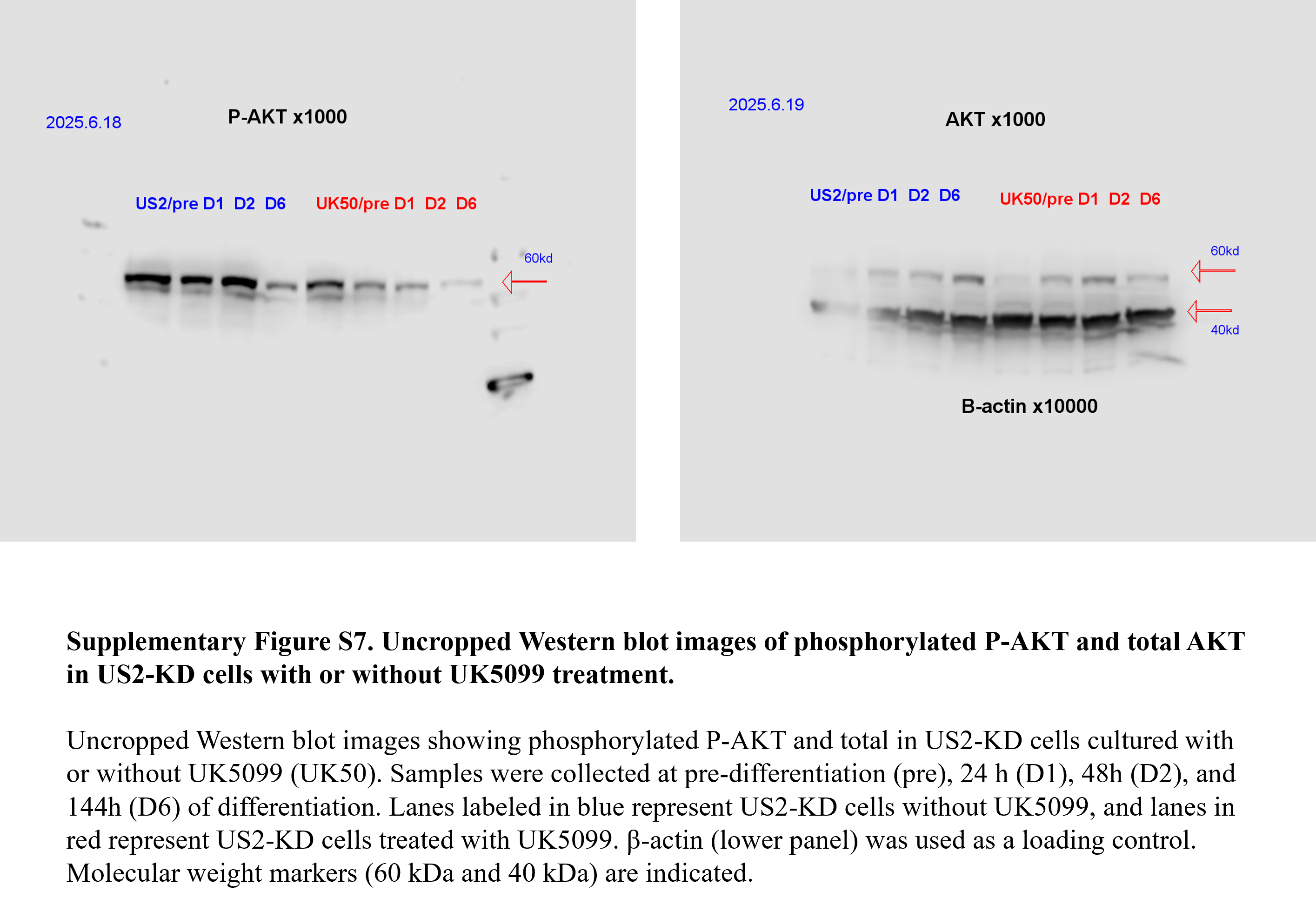

Supplement: Supplementary file 10 — Supplementary Material 10 [file 41598_2025_13764_MOESM10_ESM.tiff]
